# Young Woman With Right Lower Quadrant Abdominal Pain

**DOI:** 10.1016/j.acepjo.2024.100033

**Published:** 2025-01-13

**Authors:** Gökhan Yılmaz

**Affiliations:** Department of Emergency Medicine, Konya Meram Public Hospital, Konya, Turkey

**Keywords:** intussusception, abdominal pain, emergency department

## 1 Case Presentation

**Figure 1:**
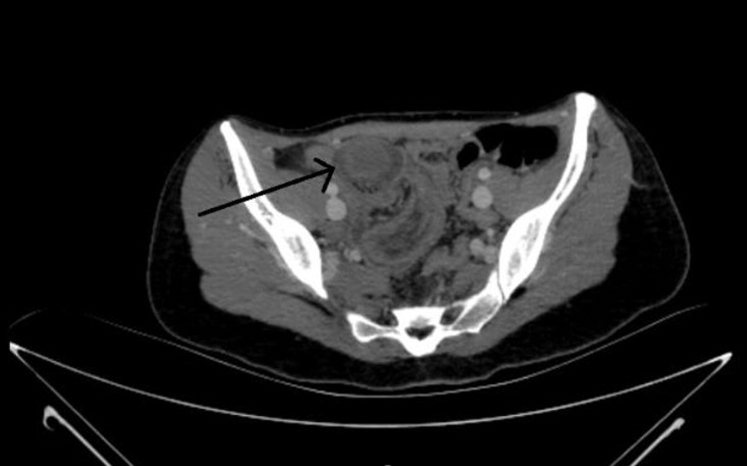
Axial view of the 3-layered structure in the ileocecal region suggesting intussusception on the patient's computed tomography (ring structure at the tip of the black arrow).

**Figure 2:**
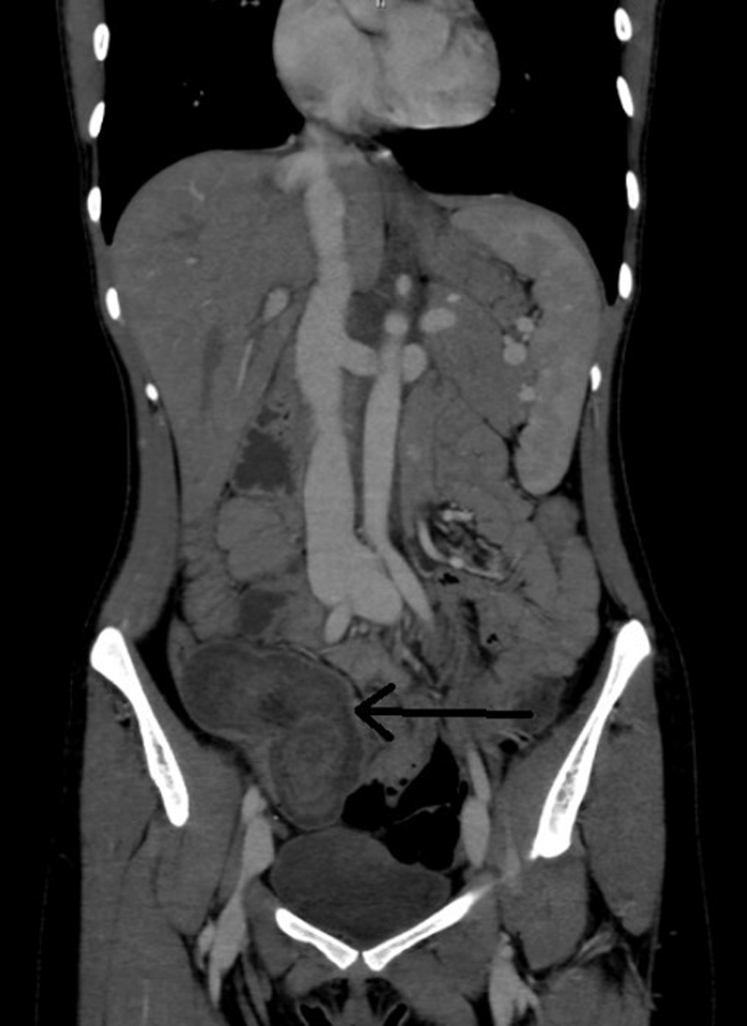
Coronal view of the 3-layered structure in the ileocecal region suggesting intussusception on the patient's computed tomography (ring structure at the tip of the black arrow).

A healthy 26-year-old woman presented with right lower quadrant abdominal pain. The pain was accompanied by bloody diarrhea and vomiting. On examination, there was tenderness in the right lower quadrant. Strawberry jelly-colored stool was detected in her rectal examination. Laboratory tests were completely normal. In our patient's contrast-enhanced computed tomography, the appearance of a triple-layered structure was detected (Figs 1 and 2).

## 2 Diagnosis: Intussusception

Intussusception is the invagination of one part of the intestine into another more distal.^1^ It is the most common cause of intestinal obstruction in infants. It usually occurs between 4 and 10 months.^1^ It can cause intestinal necrosis and death in children.^1^ Although intussusception is a common disease in infants, it is rare in adults.^2^ The presenting symptoms and signs are uncharacteristic. Therefore, the diagnosis may be missed in the emergency department.^2^ Intussusception is present in 1% of patients with intestinal obstruction.^3^ Although it is idiopathic in 90% of children, it is associated with 3/4 of the malignancies in adults.^3^ There was no underlying tumor in our patient. This made us think that it might be idiopathic.

Computed tomography with oral and intravenous contrast is the gold standard for intussusception diagnosis. Computed tomography typically shows a 3-layered structure that includes the compacted intestinal wall, its mesentery, and the surrounding intestine.^4^ This appearance was present in the computed tomography of our patient.

In conclusion, emergency physicians should consider the diagnosis of intussusception when they encounter the 3-layered structure on computed tomography.

## Funding and Support

The author received no financial support for the research, authorship, and/or publication of this article.

## Conflict of Interest

The author has affirmed he has no conflicts of interest to declare.
